# One-Step Etching Characteristics of ITO/Ag/ITO Multilayered Electrode in High-Density and High-Electron-Temperature Plasma

**DOI:** 10.3390/ma14082025

**Published:** 2021-04-17

**Authors:** Ho-Won Yoon, Seung-Min Shin, Seong-Yong Kwon, Hyun-Min Cho, Sang-Gab Kim, Mun-Pyo Hong

**Affiliations:** 1 Department of Applied Physics, Korea University, 2511 Sejong-ro, Sejong 30019, Korea; madma@korea.ac.kr (H.-W.Y.); kaisse@korea.ac.kr (S.-M.S.); rmadhrhd@korea.ac.kr (S.-Y.K.); 2 Process Research Team, Samsung Display, 1, Samsung-ro, Giheung-gu, Yongin-si 17113, Korea; h0522.cho@samsung.com (H.-M.C.); ksgking@samsung.com (S.-G.K.); 3 Stretchable Display Research Center, Korea University, 2511 Sejong-ro, Sejong 30019, Korea

**Keywords:** ITO/Ag/ITO multilayer, one-step dry etch, H_2_/HCl gas, ECR-RIE, TE-OLED

## Abstract

This paper presents the dry etching characteristics of indium tin oxide (ITO)/Ag/ITO multilayered thin film, used as a pixel electrode in a high-resolution active-matrix organic light-emitting diode (AMOLED) device. Dry etching was performed using a combination of H_2_ and HCl gases in a reactive ion etching system with a remote electron cyclotron resonance (ECR) plasma source, in order to achieve high electron temperature. The effect of the gas ratio (H_2_/HCl) was closely observed, in order to achieve an optimal etch profile and an effective etch process, while other parameters—such as the radio frequency (RF) power, ECR power, chamber pressure, and temperature—were fixed. The optimized process, with an appropriate gas ratio, constitutes a one-step serial dry etch solution for ITO and Ag multilayered thin films.

## 1. Introduction

Top-emitting organic light-emitting diodes (TE-OLED) appear suitable for high-resolution active-matrix OLED displays, due to their enhanced light outcoupling, large aperture ratio, and ease of integration with non-transparent active-matrix backplanes. TE-OLEDs commonly use a mirror-like reflective anode and a semitransparent cathode, from which the light is outcoupled. As such, Ag, Al, Au, Ni, and Pt metals used as the anode in TE-OLEDs have been found to achieve effective hole injection and light outcoupling, which are key factors [1,2,3,4,5]. Among those candidates, Ag shows high reflectance, great conductivity, and a low level of absorption. Despite these outstanding points, Ag has a low work function of ~4.3 eV, providing inefficient hole injection, and its diffusion into the organic layers severely affects light emission in an OLED device, through either plasmonic or non-radiative quenching effects [6]. The incorporation of surface treatment or a buffer layer has been reported in attempts to tailor the electronic structures of the anode/organic interface, and thus improve hole injection [7]. Capping Ag with indium tin oxide (ITO) is one of the structural changes that have been suggested in order to solve the previously mentioned problems, as well as to prevent both deterioration during contact with the bottom substrate, and the degradation of reflectivity.

In order to implement ITO/Ag/ITO multilayered thin films as the metallization layers in TE-OLEDs or electronic devices, nanopatterns or -structures with high fidelity must be generated. Although liquid solutions can facilitate high selectivity for etching processes, as the dimensions of the films in devices decrease below the micron scale, the isotropic etching process cannot control the device’s critical dimensions due to the occurrence of mask undercut, which leads to device failure [8]. To solve such problems, plasma-based dry etching has been used to produce anisotropic etch profiles, whereby the etch rate is partially controlled by chemical reactions, and enhanced by directional ion bombardment. Unfortunately, until now, only a few investigations into dry etching have been performed for ITO/Ag/ITO multilayered structures.

Silver halide produced during the dry etching process restricts the ability to dry etch Ag films. Using chlorine gas plasma, an additional wet etch step using a photoresist (PR) stripping solvent was reportedly required, resulting in undercut beneath the mask and the rough sidewall [9]. Using pure CF_4_ plasma required the fluorinated silver products to be washed out using PR stripping solvent, but residues remained [10]. An Ag etch rate of 112.6 nm/min was achieved in an electron cyclotron resonance-reactive ion beam etching (ECR-RIBE) system with CF_4_/Ar gas; however, rough surfaces and non-uniform sidewalls were observed [11]. The surface roughness of Ag film after dry etching with halogen gases has been investigated [12]. Chlorine plasma etching seems to enhance the compressive stress of the Ag film, resulting in increased boundary grooving and agglomeration. In contrast, fluorine plasma etching resulted in reduced Ag film stress, and suppressed the surface roughness. To avoid the halogen reaction process, the silver film was oxidized using an O_2_ plasma process and removed by an H_2_O_2_ wet process. However, the PR mask degraded when exposed to the O_2_ plasma, causing a non-uniform pattern [13].

Silver film etching using H_2_ plasma at 200–400 °C has been reported, but the etch rates are uncertain [14]. H_2_ plasma succeeded in etching Ag films at 33 nm/min using inductively coupled plasma (ICP) under conditions of 10 °C, 100 W (platen)/500 W (coil), an H_2_ flow rate of 50 sccm, and 20 mTorr working pressure [15]. The Ag etch rate was increased by the effects of ion bombardment, but also by photon enhancement and by reactive chemistry. The Ag dihydride anion is more thermodynamically stable than the compositional elements, and therefore represents a possible product species, while neutral Ag hydride is unstable relative to its metal and hydrogen gas components [16]. Silver’s surface morphology when reacted with various plasmas (argon, hydrogen, nitrogen, air, and oxygen plasma) has been analyzed and compared [17].

The plasma etching of ITO films has been reported, using a capacitively coupled plasma as well as an ICP and an ECR plasma, where halogen gases and organic gases were used. In the case of halogen gases, such as CCl_4_ and HCl, it is comparatively easy to obtain a high etch rate of ~70 nm/min, forming InCl_3_ and SnCl_4_ as etch products, while the selectivity to silicon dioxide or silicon nitride is relatively low [18,19]. Investigation of the cleaning of the chamber wall after the chlorine plasma etching of ITO films provided promising results for maintenance [20]. On the other hand, the opposite tendencies—that is, a low etch rate and high selectivity—are found for organic gases such as CH_4_/H_2_, C_3_H_6_O, and CH_3_OH, used for forming dimethyltin (C_2_H_6_Cl_2_Sn) and tetramethyltin (C_4_H_12_Sn) as etch products. Etching and deposition occur simultaneously with the use of an organic etching gas, and this is not desirable in most cases, either from the standpoint of the part being etched, or in terms of keeping the reactor chamber clean [21,22,23,24].

In general, a high-density, high-electron-temperature plasma is desirable for the high-speed etching of metal electrodes. Here, we present the etching process for a high-density and high-electron-temperature plasma source, using an 875 G magnetic field and 2.45 GHz microwave propagation on the bulk plasma, whereat a process of ECR occurs with a H_2_/HCl gas system. Although HCl is not selective towards any underlying silicon, this study is designed to develop a one-step etch process for multilayer thin films that consist of ITO and Ag thin films.

## 2. Materials and Methods

**Figure 1:**
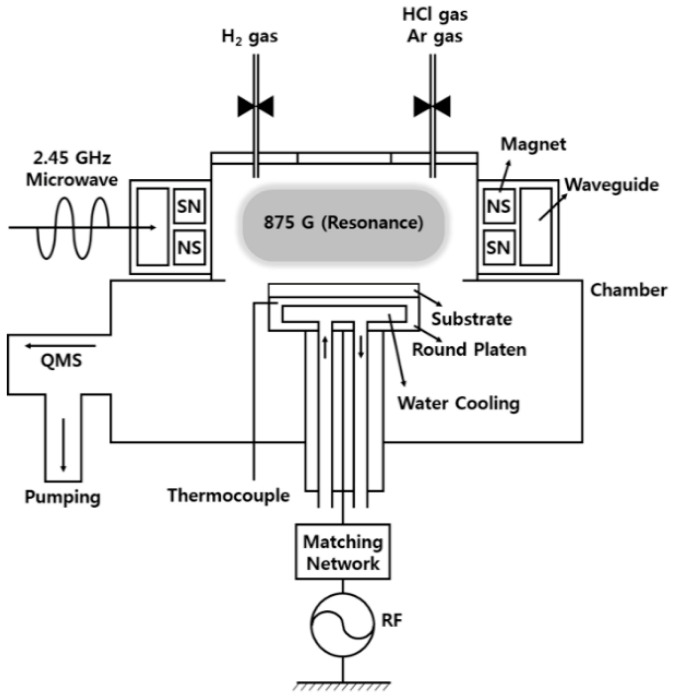
Schematic diagram of an electron cyclotron resonance-reactive ion etching (ECR-RIE) system for indium tin oxide (ITO)/Ag/ITO multilayered thin film.

In this experiment, we used a cylindrical ECR plasma source, surrounded by magnets installed at intervals of several millimeters so as to provide the 875 G magnetic field, while a 2.45 GHz microwave was introduced using a waveguide in order to increase the plasma density, as shown in Figure 1 [25,26,27]. The substrates (samples) were placed on a round platen designed for 13.56 MHz radio frequency (RF) self-bias and water cooling. During the etching process, the ECR power and RF bias power were fixed at 900 W and 300 W, respectively. The total gas flow was 60 sccm, and the operating pressure was 5 mTorr. The self-bias decreased from −400 V—at 100% HCl—to a minimum of −310 V, according to the increase in H_2_ concentration. To prevent the vaporization of non-volatile products during the process, the substrate heated by the ECR plasma was cooled to maintain a temperature under 80 °C, and was monitored via a thermocouple placed in the side of the round platen.

For the preparation of the ITO/Ag/ITO multilayered samples, we used magnetron sputtering. The base pressure of the sputter was 5.0 × 10^−6^ Torr, and the working pressure was 6 mTorr with 20 sccm of Ar gas (99.999%) introduced to the chamber. During sputtering, the substrate was rotated at 15 rpm to ensure uniform thickness. The first layer-ITO-was 100 Å thick, and was deposited with a target consistency of 90 wt.% In_2_O_3_ and 10 wt.% SnO_2_ onto a glass substrate. The second layer-Ag-was deposited at a thickness of 1000 Å, with a target purity of 99.99% Ag, while the last layer-ITO-was deposited at a thickness of 100 Å without breaking the vacuum. To evaluate the etching characteristics of ITO and Ag, 3000 Å single-layer deposits were prepared separately.

## 3. Results and Discussion

This section is divided into two parts: First, the etching characteristics of samples with single-layered ITO and Ag thin films are investigated. Second, based on these findings, the etching process for multilayered ITO/Ag/ITO thin films is optimized. Since the bombardment of ions onto the substrate during a long etching process may increase the temperature and cause changes in the etch chemistry, water backside cooling was carried out in all of the dry etching experiments.

In this work, the etching process is based on the use of H_2_ and HCl gases. The plasma chemistry of HCl has similar properties to that of Cl_2_ + H_2_; however, it also produces chlorine and hydrogen ions at a ratio of 1:1 during dissociation, which makes its use a more efficient approach than breaking high-energy bonding hydrogen molecules. Higher H ion bombardment helps to break the oxygen bonds on the ITO surface, and the consequential chemical reactions produce volatile etch products, such as OH [28,29,30]. In addition, this process makes the film more accessible, and makes the surface more reactive to the reactive species of chlorine. The ITO etch rates achieved with the combination of H_2_ and HCl are greater than the etch rates obtained with the same fluxes used separately. For Ag etching, the excessive reactivity of H is essential for balancing the reactivity of Cl, which interrupts the etching process and causes the growth of AgCl nanorods. It is reasonable to remove the chlorine gas during the Ag etching process; however, this lowers productivity due to the increased process time required by the multiple steps. Therefore, an optimized combination of H_2_ and HCl is required for the etching of the ITO/Ag/ITO multilayered structure in a one-step process, without changing etching conditions.

### 3.1. Single ITO and Ag Layers

Changing the ratio of these gases should directly affect the etching characteristics. The etching behaviors of single-layered ITO and Ag thin films were investigated, by keeping the total gas flow rate constant at 60 sccm, and varying that of H_2_ from 0 to 60 sccm in the H_2_/HCl gas mixture. To verify the role of the H_2_ gas in the ITO etching process, etching experiments with H_2_ and Ar were also conducted, and their results were compared with those for the H_2_ and HCl gases. The etch rate was estimated from the depth of the etched samples, and this depth was measured using a surface profiler (KLA-Tencor Alpha-Step D-600, Milpitas, CA, USA).

**Figure 2:**
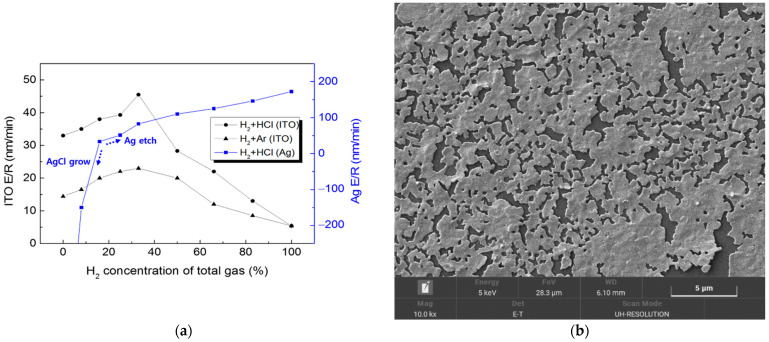

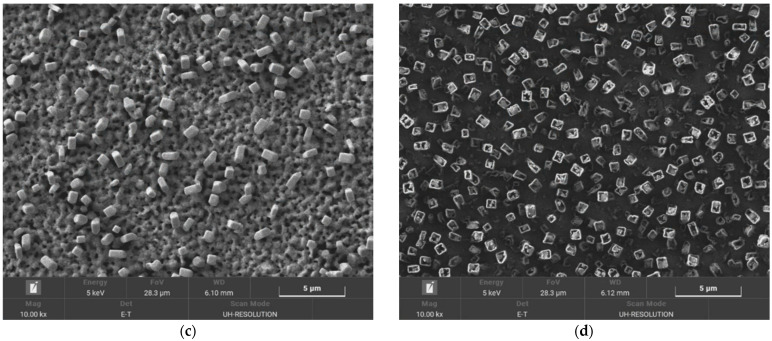
(**a**) Etch rate of ITO and Ag single-layered films, assessed following changes in H_2_ concentration in the total gas flow. (**b**) Scanning Electron Microscope (SEM) micrographs of etched surfaces of Ag. (**c**) The grown AgCl nanorods, and (**d**) the AgCl nanotubes.

Figure 2 shows that ITO achieved the highest etch rate peaks at a 33% concentration of H_2_ under the H_2_ + HCl and H_2_ + Ar gas environments. It is evident that the ITO etching process here was dominated either by reactive chlorine chemical etching or by the physical Ar ions’ etching, while the hydrogen ions enhanced etching speed by breaking the oxygen bonds on the ITO surface. For H_2_ concentrations of over 50%, the number of reactive species in the plasma was insufficient to etch out the entire exposed ITO surface, chemically or physically. HCl is preferred over Ar for achieving a higher etch rate, and for suppressing In redeposition. For Ag etching, with a H_2_ concentration of less than 17%, the chlorine overwhelmed the hydrogen to form AgCl products, which grew gradually from nanodots to nanorods and hollow nanotubes. With an H_2_ concentration over 17%, the etching process was dominated by reactive hydrogen over chlorine, which successfully suppressed the AgCl. The Ag etch rate increased with a more highly concentrated H_2_ flow, forming volatile silver hydrides. With an H_2_ concentration over 50%, the Ag etch rate reached over 100 nm/min, which is four times faster than the ITO etch rate. Ag etching using H_2_ + Ar gases was easily achieved; however, due to the redeposition of the sputtered Ag all over the inner components of the chamber, it was not repeated with varying concentrations of gases.

**Figure 3:**
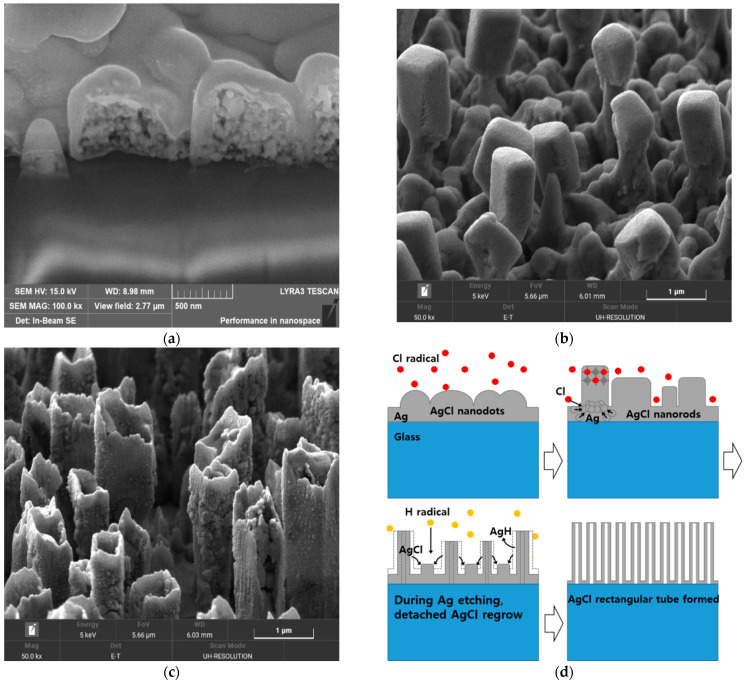
SEM images of a multistep dry etched Ag single layer. (**a**) In step 1, an ECR-RIE system containing HCl is used to convert Ag to AgCl nanodots, and (**b**) they grow as nanorods. (**c**) In step 2, H_2_ plasma alone is used to attempt to vaporize AgCl nanorods, which become hollow rectangular nanotubes. (**d**) Diagrams explaining the transition of AgCl nanorods to nanotubes, and their increased density. During H_2_ plasma etching, hydrogen reactively vaporizes the Ag present in the mixed Ag/AgCl nanorods, and the AgCl produced by Ag vaporization re-attaches onto the surface of the substrate and regrows as nanorods, until only the AgCl remains, in nanotube form.

In order to clarify the roles of hydrogen and chlorine, and to see if the AgCl that grew could be etched, a two-step etch process was investigated, and its results were monitored using Scanning Electron Microscope (SEM, MIRA3, Tescan, Brno, Czech Republic) micrographs, as shown in Figure 3. Firstly, Ag was reacted with the HCl plasma to form AgCl, and secondly, the H_2_ plasma was exposed to the grown AgCl in an attempt to vaporize it. The AgCl nanorods were partially etched, and adopted hollow rectangular tubular shapes during exposure to the H_2_ plasma, but the density of the AgCl also increased, filling the spaces between the AgCl tubes. It could be hypothesized that, during the second step, the hydrogen plasma vaporizes the Ag present in the mixed Ag/AgCl nanorods, and the AgCl produced by the Ag vaporization re-attaches to the surface of the substrate, where it regrows as nanorods until only AgCl is left, in a hollow nanotube form. Therefore, the AgCl that is grown is difficult to etch out consecutively with hydrogen plasma, and it is necessary to suppress the AgCl formation from the beginning. In order to prevent the AgCl from growing, or the sidewall from being damaged, during the ITO/Ag/ITO etching process, it is appropriate to control the H_2_ concentration in the gas mixtures at between approximately 17% and 33%.

### 3.2. ITO/Ag/ITO Multilayers

**Figure 4:**
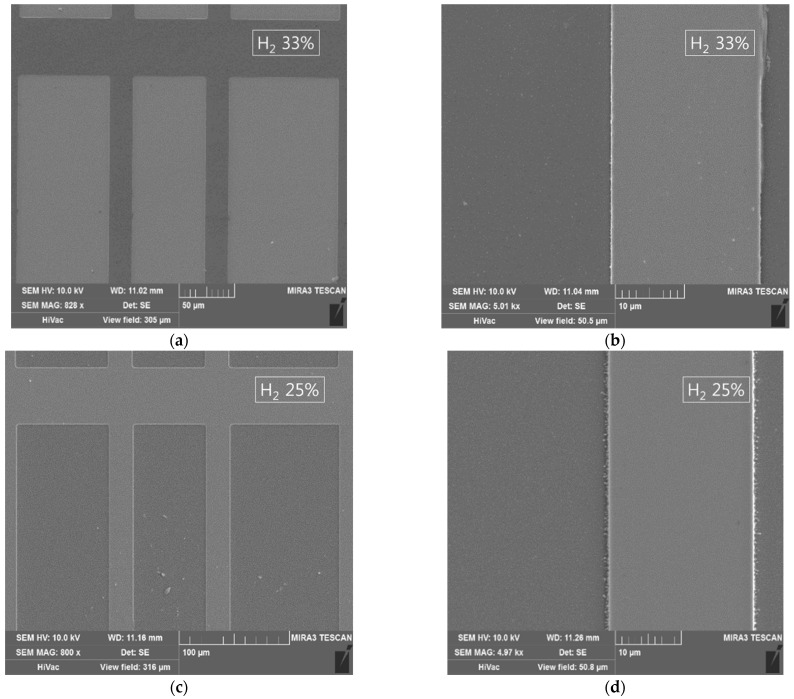

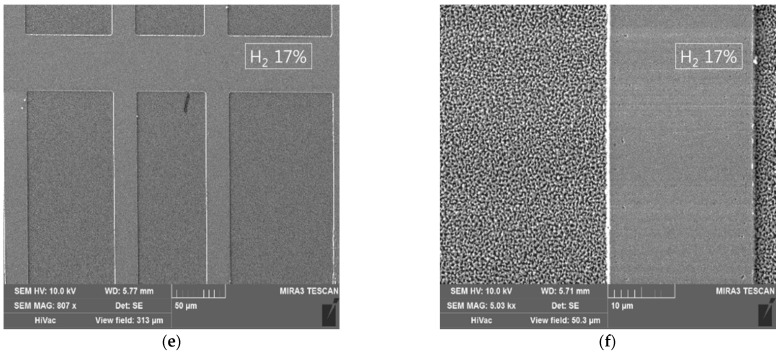
SEM images of dry etched ITO/Ag/ITO using an ECR-RIE system containing the H_2_ and HCl gases with optimized H_2_ concentration. (**a**,**b**) A 33% concentration of H_2_ manifests a clean etch profile without any residue. (**c**,**d**) A 25% concentration of H_2_ results in little residue being left on the edge or sidewalls. (**e**,**f**) A 17% concentration of H_2_ results in the growth of AgCl during the process and residue being left all over the etched area.

**Figure 5:**
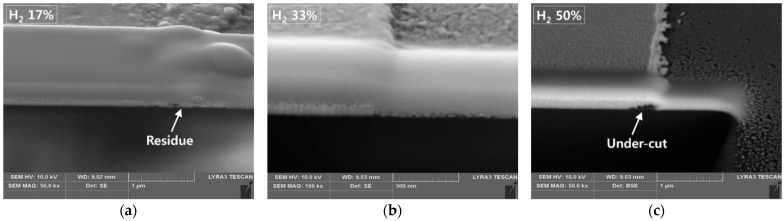
Cross-section of the etched profile using different H_2_ concentrations. (**a**) The etch process with insufficient hydrogen etch results in residue left at the edges of patterns. (**b**) The process with optimized hydrogen concentration causes neither residue nor undercutting. (**c**) Oversupplying hydrogen leads to undercutting.

For the one-step etching process for the ITO/Ag/ITO multilayered electrode, the concentrations of H_2_ in the H_2_ and HCl gas aspects were kept between 17% and 33%. Figure 4 shows SEM micrographs of ITO/Ag/ITO etched using the 17%, 25%, and 33% H_2_ concentrations. Several 50 um scale images are presented to show the size of the pattern (21 um width lines separated in 50, 75, and 100 um pitches). Because the differences between the samples were not clearly distinguishable at a large scale, 10 um scale images are added to show the remains left over the etched area at lower concentrations of hydrogen gas. A 33% concentration of H_2_ manifested a clean etch profile with smooth sidewalls. The absence of any undercutting in the etch profile suggests that both the ITO and the Ag were etched at an appropriate rate (1:1.8). The 25% concentration of H_2_ also manifested a clean etch profile, with approximately the same etch rate (1:1.3); however, some residues were left on the edges. With the 17% concentration of H_2_, the insufficient presence of hydrogen ions or reactors caused more residue to be left on the substrate surface, with an Ag etch rate lower than that of the ITO (1:0.8). Figure 5 shows the cross-sections of the etched profiles of ITO/Ag/ITO using 17%, 33% and, 50% H_2_ concentrations taken by Focus Ion Beam Scanning Electron Microscope (FIB-SEM, LYRA3, Tescan, Brno, Czech Republic). The slow Ag etching speed causes residue to be left even if the processing time is increased up to the point at which the etched profile is damaged by the ITO’s side etch. The etching process with an H_2_ concentration of over 50% led to undercutting between the ITO and Ag layers, or over-etched patterns due to the higher etch rates of Ag thin film (4–31 times faster). The optimized balance led to an appropriate profile without residue or undercutting.

**Figure 6:**
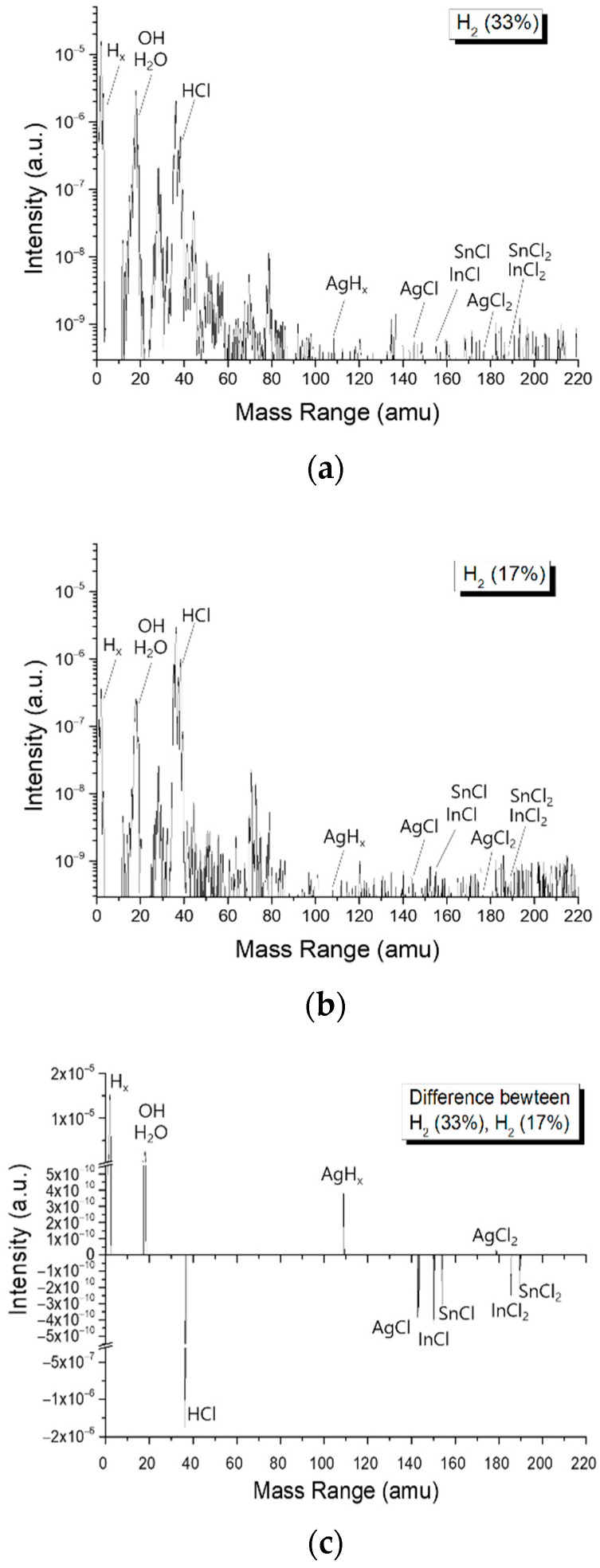
Mass spectra for (**a**) H_2_ 33%, (**b**) H_2_ 17% in the H_2_ + HCl etching process, and (**c**) the difference in the intensity between (**a**,**b**).

The gases produced through the etching process were measured in the range of 1–220 amu, with a mass separation of ~0.16, with a quadrupole mass spectrometer (QMS, Dycor System 200 LC-D, Newark, DE, USA) mounted on a port of the etching chamber. Figure 6a,b shows the intensities of the mass spectra for the etching processes with 33% and 17% concentrations of H_2_, respectively, using the same recipe and the same ITO/Ag/ITO sample, while (c) shows the difference between (a) and (b). With the 33% H_2_ gas process, a large quantity of hydride compounds was observed; on the other hand, with the 17% H_2_ concentration process, the presence of hydride compounds decreased, while that of halide compounds increased. Similar to the QMS results, the redeposition onto and/or contamination of the chamber walls was greatly reduced as the H_2_ concentration increased.

## 4. Conclusions

In this work, an H_2_/HCl-based one-step dry etching process for a sputtered ITO/Ag/ITO multilayered electrode has been developed. By investigating the effect of a combination of H_2_ and HCl on single-layered ITO and Ag thin films, the etching process has been optimized. Using HCl as the etching gas—instead of Cl_2_ or H_2_—facilitated the efficient supply of reactive hydrogen radicals in a ratio of 1:1 with chlorine, and increasing amounts of H_2_ were added to supply a greater amount of hydrogen radicals, so as to prevent AgCl from growing and to ensure the successful etching of Ag. The ITO single layer was dry etched with pure HCl, and added H_2_ gas enhanced the etch rate. Exceeding 50% H_2_ concentration, the ITO etch rate was decreased due to oversupplied hydrogen and insufficient chlorine. On the other hand, the Ag single layer was easily dry etched with pure H_2_ using the high-temperature plasma. There is no reason to add any chlorine in order to etch Ag; however, in order to dry etch ITO/Ag/ITO multilayers in a one-step process, the effect of chlorine gas on the Ag dry etching process was observed. The Ag etch rate kept decreasing as HCl gas concentration was increased to 80%. AgCl began to grow when the HCl concentration exceeded 90%. We expected that the ITO/Ag/ITO multilayered film would follow the etching trend of ITO and Ag single layers, and it seemed appropriate to maintain the H_2_ concentration in the H_2_ + HCl gas mixtures at between approximately 17% and 33% in the ECR-RIE system. The optimal concentration of H_2_ in its balance with HCl gas is 25–33%, through which a smooth, vertical, and residue-free etch profile of the ITO/Ag/ITO multilayered electrode has been achieved. We suggest that the concentration of H_2_ should be modified if the etch rate difference between Ag and ITO is changed, according to the conditions of the etching environment.

## Data Availability

The data presented in this study are available on request from the corresponding author. The data are not publicly available due to legal issues.
